# The genetic diversity and differentiation of mussels with complex life cycles and relations to host fish migratory traits and densities

**DOI:** 10.1038/s41598-020-74261-z

**Published:** 2020-10-15

**Authors:** Martin Österling, Manuel Lopes-Lima, Elsa Froufe, Amra H. Hadzihalilovic, Björn Arvidsson

**Affiliations:** 1 Department of Environmental and Life Sciences – Biology, Karlstad University, Universitetsgatan 2, 651 88 Karlstad, Sweden; 2 CIIMAR/CIMAR - Interdisciplinary Centre of Marine and Environmental Research, University of Porto, Terminal de Cruzeiros do Porto de Leixões, Av. General Norton de Matos s/n, 4450-208 Matosinhos, Portugal

**Keywords:** Genetic variation, Conservation biology, Freshwater ecology

## Abstract

Many landscape and biotic processes shape the genetic structure of populations. The genetic structure of species with parasitic stages may also depend on the life history and ecology of their host. We investigated population genetic structure of the mussel *Margaritifera margaritifera* in Southern Sweden, and in relation to the population size and life history of its hosts, *Salmo trutta* and *S. salar*. Mussel populations were genetically differentiated into two clusters, further subdivided into four clusters and distinct conservation units. Regardless of host species, the genetic differentiation was lower among mussel populations sustained by sea-migrating than by resident hosts, while the genetic diversity was higher in mussel populations sustained by sea-migrating than by resident hosts. Genetic diversity of mussel populations was positively related to host abundance. Mussel population size was positively related to high genetic diversity of mussels sustained by resident hosts, while low mussel population size sustained by sea-migrating hosts had a high genetic diversity. The results of our study suggest a combined influence of mussels and host fish on genetic structure of unionoid mussels. We suggest to conserve not only mussel population sizes and host fish species, but also consider host migratory/resident behaviour and abundance when designing conservation programs.

## Introduction

Several historic processes shape the structure of landscapes, with consequences for the genetic structure of populations at local and regional scales^1–3^. Recent anthropogenic processes such as habitat destruction and fragmentation often lead to reduced population sizes and connectivity, reducing the genetic diversity and increasing the genetic differentiation^4^. In addition, life history traits and species interactions affect the genetic structure of populations at different spatial and temporal scales^5^.

Species with parasitic life stages are dependent on the life history and ecology of the host, where differences in mobility patterns of the host may influence the genetic structure of post-parasitic life stages^6–9^. Such processes may result in differences in the genetic structure of post-parasitic sedentary population, depending on the host species, but also among strains of the same host species^10,11^. Population size is another factor affecting the genetic structure, where a high population size and effective population size, respectively, often positively affects genetic diversity^12^. In addition, the host population size is a factor that potentially affects the genetic structure of species with parasitic life stages. Many host individuals may increase the chance for each individual parasite to infest a host and pass on their genes to the next generation, sustaining the genetic diversity of the post-parasitic population. Mobile and dense host populations might thus increase the potential for high numbers of spawning parasitic individuals infecting their hosts, and connect sub-populations of post-parasitic populations, thereby sustaining the genetic diversity and decreasing the genetic differentiation.

The threatened unionoid mussels are obligate parasites on one or more fish species, and the mobility pattern of the host fish is one factor affecting the distribution and passive movement of the mussels^13^. Combined with the connectivity within and among river systems, host fish mobility may thus affect the genetic structure of mussel populations. Mussel populations sustained by host fish species with a high mobility may thus show less differentiation and higher genetic diversity compared to mussel populations sustained by stationary host fish species^10,14^. Such genetic structure may also be more accentuated between areas with high versus areas with low connectivity between mussel populations. In addition, population sizes of host fish typically differ manifold among mussel species and populations^15^, with potential consequences on the genetic diversity of the mussel populations. Thus, the higher the host fish population size and abundance, the higher potential for high genetic diversity of parasitic mussel larvae on the fish, and consequently for the mussels leaving the host fish entering the benthic mussel population.

The threatened freshwater pearl mussel (*Margaritifera margaritifera*) is parasitic on Atlantic salmon (*Salmo salar*) and/or brown trout (*Salmo trutta*) for 10–12 months, whereupon they fall off the fish and become sedentary mussels^16^. Atlantic salmon, with their high abundances of young-of-the-year (YOY) individuals, is generally considered as the primary host for the mussel in river reaches where their distribution overlap. Sea-migrating brown trout, also with high abundances of YOY individuals, appear to be the host in river reaches where they spawn and Atlantic salmon has no historical distribution. Finally, in tributaries higher up in river catchments, tributary resident brown trout, with comparatively low abundances of YOY individuals, are functional hosts^10,17–20^.

Population genetic studies of the freshwater pearl mussel have shown high genetic differentiation among river drainages, but also among populations independent of drainages^21^. One exception is the North American distribution, with its low differentiation among populations^22^. The lowest genetic diversity occurs in the mussel’s southwestern distribution along the Atlantic coast in Iberia^21^. In contrast, the genetic diversity is higher along the Atlantic coast of the British Isles and western Scandinavia^10,14^. The genetic diversity is somewhat lower in central Europe, while it is higher in areas in mid- and northern Baltic Sea drainages^23^.

Regarding host fish species, some interesting patterns have also been found, i.e., mussel populations using Atlantic salmon as hosts show lower genetic differentiation and higher genetic diversity, compared to mussel populations using brown trout as host fish^10,24,25^. These patterns are related to the fact that the population size of mussels has been suggested to be positively related to genetic diversity, with high mussel population size in Atlantic salmon rivers and low mussel population size in brown trout rivers^14^. However, there is a lack of knowledge of the general effects of migratory/residence life history of host fish, and the size and abundance of host fish populations on genetic structure of mussel populations. Sea-migrating brown trout have been shown to have higher genetic diversity and lower genetic differentiation among their populations compared to tributary resident brown trout populations^11^. Given that the movement of the mussel is tightly connected to their host fish, this leads us to believe that the patterns of genetic structure of mussel populations is not solely dependent on fish species, but also on their migratory/residence life history, i.e. sea-migrating brown trout and Atlantic salmon vs. tributary resident brown trout. The genetic diversity may thus be higher and genetic differentiation lower in mussel populations sustained by sea-migrating brown trout and Atlantic salmon compared to mussel populations sustained tributary resident brown trout.

The overall aim of the present study was to investigate population genetic structure, i.e. genetic differentiation and diversity, in seven drainages with two host fish species with different inter- and intraspecific patterns of mobility, i.e. Atlantic salmon, sea-migrating brown trout and tributary resident brown trout. We hypothesize that (1) populations are genetically differentiated among drainages (2) the genetic differentiation is higher between mussel populations sustained by tributary resident brown trout compared to mussel populations sustained by sea-migrating brown trout and Atlantic salmon (3) the genetic diversity is higher in mussel populations sustained by sea-migrating brown trout and Atlantic salmon compared to mussel populations sustained by tributary resident brown trout. We also hypothesize that the genetic diversity increase both as a function of mussel population size and host fish abundance. Based on this knowledge, one last aim is to recommend management strategies and conservation programs.

## Materials and methods

### Study area, sampling, and DNA extraction

**Figure 1:**
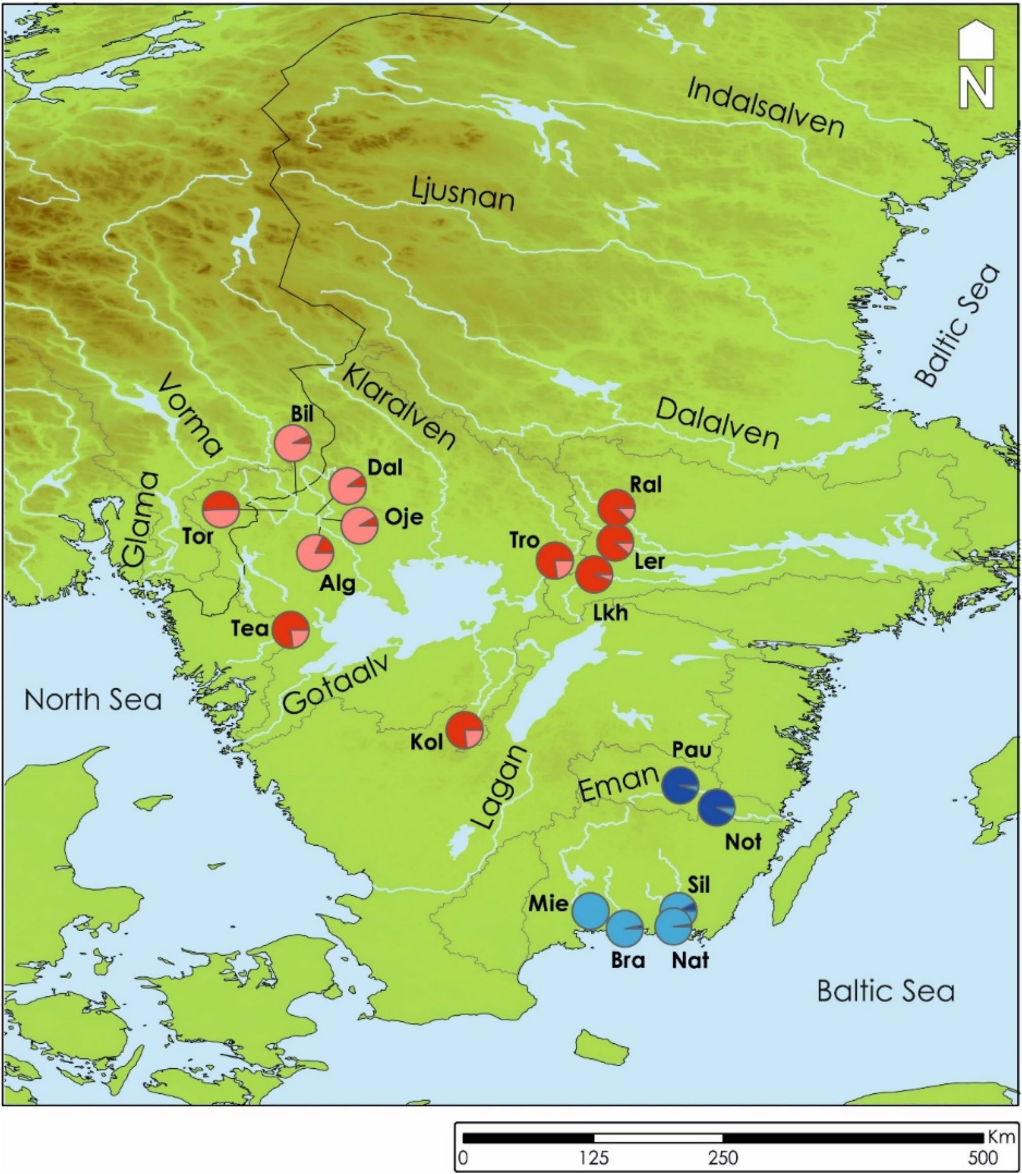
Map showing the distribution of the sampled freshwater pearl mussel populations. Pie charts represent the average % assignment of individuals (based on membership coefficient) into each of the four recognized Conservation Units (see Fig. 2 for color codes). ArcMap 10 Environmental Systems Resource Institute (ESRI) (2010).

A total of 732 freshwater pearl mussel specimens were collected in 2005 from seventeen populations from seven main drainages in Southern Sweden. In eleven of these populations, tributary resident brown trout populations are functional hosts, while four mussel populations are known to have sea-migrating brown trout as hosts. For the two remaining mussel populations, sea-migrating brown trout and Atlantic salmon had overlapping distribution before the hydropower plants blocked their migration. Probably Atlantic salmon is the natural host for the mussels in these two populations, as this has been shown when the distribution of Atlantic salmon and the freshwater pearl mussel overlap^18^ (Fig. 1, Table 1).

**Table 1:** Location of the analysed freshwater pearl mussel populations and corresponding microsatellite statistics; *N* = number of samples; *N*a = number of observed alleles per population; *N*_PA_ = Number of Private alleles per population; *H*_E_ = mean expected heterozygosity; *H*_O_ = mean observed heterozygosity; *F*_IS_ = mean inbreeding coefficient; and *HWE* = Hardy–Weinberg deviation (*P*).

Population	Code	River drainage	Latitude	Longitude	N	N_A_	N_PA_	H_E_	H_O_	F_IS_	HWE
**Sea-migrating brown trout host**											
Silletorpsån	Sil	Silletorpsån	56.323483	15.587049	48	26	1	0.61	0.51	0.17	< 0.01
Nättrabyån	Nat	Nättrabyån	56.189764	15.544808	21	28	1	0.64	0.53	0.20	< 0.01
Bräkneån	Bra	Bräkneån	56.170012	15.123165	44	30	0	0.56	0.56	0.02	0.015
Mieån	Mie	Mieån	56.322039	14.871870	5	13	0	0.31	0.32	0.15	0.867
**Atl. salmon/sea-migrating brown trout host**											
Pauliström	Pau	Emån	57.409560	15.603895	50	32	4	0.51	0.46	0.11	< 0.01
Nötån	Not	Emån	57.218951	15.917775	48	42	11	0.60	0.52	0.15	< 0.01
**Resident brown trout host**											
Billan	Bil	Göta älv	59.915514	12.277729	50	16	0	0.34	0.16	0.52	< 0.01
Dalsälven	Dal	Göta älv	59.757485	12.438663	50	13	0	0.31	0.17	0.44	< 0.01
Torgilsrudsälven	Tor	Göta älv	59.813611	12.187733	50	14	0	0.18	0.08	0.57	< 0.01
Älgån	Alg	Göta älv	59.643816	12.479883	50	17	0	0.34	0.13	0.62	< 0.01
Öjenäsbäcken	Oje	Göta älv	59.753423	12.480199	50	15	1	0.31	0.14	0.56	< 0.01
Lerkesån	Ler	Norrström	59.511150	15.040410	51	31	4	0.55	0.35	0.38	< 0.01
Lekhytteån	Lkh	Norrström	59.235893	14.859130	50	19	1	0.19	0.10	0.50	< 0.01
Rällälven	Ral	Norrström	59.811661	15.052239	48	25	2	0.45	0.33	0.30	< 0.01
Trösälven	Tro	Göta älv	59.355954	14.517684	48	24	0	0.35	0.19	0.48	< 0.01
Kolarebäcken	Kol	Göta älv	57.890289	13.660799	33	21	0	0.40	0.36	0.14	< 0.01
Teåker	Tea	Göta älv	58.757500	12.231099	36	26	0	0.53	0.47	0.17	< 0.01

Two principal sources for DNA extractions were used in this study: biopsies of foot tissue, both from freshly dead and living specimens, and sampling of haemolymph from living specimens. For the first method, mussels were induced to open their valves by putting them in 30 °C water, a sample of foot tissue was collected using heat sterilized tools^26^. Those samples were stored in 70% ethanol and kept cold refrigerated at 8 °C until DNA was extracted. For the latter method, mussels were removed from the river bottom and approximately 0.1–0.3 ml of haemolymph was collected with 1 ml syringes attached to 0.80 × 50 mm 21Gx 2″ sterile needles by gently inserting the needle into the foot of the mussels^23^. All mussels were then returned to their original locations within the riverbed substrate. Haemolymph samples were transferred to 1.7 ml Eppendorf vials, cooled at 5 °C and processed immediately in the laboratory. After centrifugation at 14,000×*g* for 5 min, the supernatant was discarded. DNA from foot tissue and haemolymph was isolated using a Macherey–Nagel genomic DNA from cells and tissue kit, following the company's protocol.

### Microsatellite selection and amplification

Thirteen published microsatellite loci^27^ were tested on randomly chosen individuals from different populations to determine which microsatellites generate polymorphic bands. Of these, the six microsatellite loci with the highest polymorphism level were selected for this study: MarMa 5167, 4277, 4143, 4315, 3621 and 3116. Polymerase chain reactions (PCRs) were performed in a total volume of 10 µl, with the following components: 2 µl DNA, 0.35 µl 2.5 mM dNTP, 1 µl 10XB and 0.075 µl Taq DNA polymerase (with MgCl_2_ in buffer) and different amounts of primers (from 0,1 to 0,4 µl). Two different PCR reactions were used for the 6 microsatellites. TD 55–45 for MarMa 5167, 4277, 4143 and 4315 and TD 60–50 for MarMa 3621 and 3116. PCR was done with an initial denaturation in 96 °C for 1 min, followed by 30 cycles of 96 °C for 20 s, 50 °C for 20 s and 60 °C for 4 min, and extension in 72 °C for 1 min. PCR fragments were separated using polyacrylamide gels and were analyzed in CEQ 8000 Genetic Analysis System (Beckman Coulter).

### Microsatellite analyses

All individuals were genotyped for the 6 loci, following^27^. Number of observed (*N*_A_) and private alleles (*N*_PA_), observed (*H*_O_) and expected heterozygosity (*H*_E_), inbreeding coefficients (*F*_IS_), and deviations from the Hardy–Weinberg equilibrium (HWE) were determined in GENEPOP v.4.1^28,29^. Significance exact tests were estimated by a Markov chain method after 10,000 randomizations. Presence of null alleles was tested for each locus using MICROCHECKER^30^.

Global genetic differentiation among populations was assessed with the *F*_ST_ fixation index^31^ in FSTAT 2.9.3.2^32^ with significance being assessed with 1.000 permutations with adjustment for multiple comparisons using the sequential Bonferroni method. The impact of null alleles on *F*_ST_ estimation was assessed by comparing *F*_ST_ estimates before and after correction for null alleles. Null alleles were found to have a minimal or none impact on *F*_ST_ estimates and later analysis, hence all subsequent analyses were conducted on data uncorrected for null alleles. For a tree-based analysis, *F*_ST_ were calculated and used with PopTree2^33^ to produce a Neighbour-Joining (NJ) tree based on population allele frequencies.

Population structure was analysed using the Bayesian model-based clustering approach implemented in STRUCTURE v.2.3.3^34^. Correlated allele frequencies, an admixture model without prior population information for individuals, was assumed. Fifteen independent runs were made for K = 1–20 with each run consisting of a burn-in of 10^5^ Markov-chain Monte Carlo steps, followed by 5 × 10^5^ steps. Selection of the most likely number of genetic clusters (K) was based on the second order rate of change in probability between successive K values as described in^35^ and implemented in STRUCTURE HARVESTER^36^. In systems with hierarchical population structure, STRUCTURE typically best resolves the highest level of population subdivision^35^. To resolve lower levels of subdivision, STRUCTURE analyses were also conducted separately for each of the main clusters identified (see “Results”). For this purpose, two additional STRUCTURE analyses, within each of these two clusters, were used to identify potential further structure. The same settings were used for both clusters (K = 1–10 and K = 1–15). These analyses included populations with assignment of individuals higher than 85%. Again, the method by^35^ was used to identify the most likely number of genetic clusters. Structure population analyses were further analysed using Discriminant Analysis of Principal Components with DAPC, Adegenet package in R^37,38^.

Tests for genetic differentiation between the clusters and groups identified in STRUCTURE and DAPC analyses were conducted using AMOVA in ARLEQUIN v.3.5.1.3^39^. Genetic variation within clusters (*F*_ST_), among populations within clusters (FSC) and among populations (FCT) was assessed, and significance of F-statistics was tested using 10,000 permutations.

To infer for recent demographic changes, the moment-based method of^7^ as implemented in the BOTTLENECK software was applied, assuming a two-phase mutation model (TPM)^40,41^. TPM was parameterized with 70% single-step mutations, assuming a conservative variance among multiple steps of 30, with the significance being assessed using Wilcoxon sign-rank test with 10,000 iterations. Rates of recent migration (m) among populations/clusters identified in STRUCTURE, as well as its direction, were estimated using the Bayesian multilocus genotyping procedure implemented in BAYESASS v.3.0^42^. The program was run after 10^6^ MCMC iterations, with a burn-in of 10^5^ iterations and a sampling frequency of 1,000 with default values for all parameters.

### Mussel population size and host fish density

To test our hypothesis that migratory and resident life history of the host fish can influence the genetic diversity of the mussel populations, the observed (*H*_O_), and expected (*H*_E_) heterozygosity, and inbreeding coefficient (*F*_IS_) were compared between mussel populations having sea-migrating brown trout or Atlantic salmon as host fish and mussel populations having a tributary resident brown trout population as host fish, using Mann-Whiney U-tests.

As we hypothesized that host fish abundance positively affects the genetic diversity of the mussel populations, mean brown trout abundances over thirty-one years (1986–2005) were extracted from the Swedish electrofishing database. To see if the abundance of YOY brown trout, the abundance of brown trout older than one year (1 +) and mussel population size were related to observed (*H*_O_) and expected (*H*_E_) heterozygosity, and inbreeding coefficient (*F*_IS_), stepwise regressions were run for these genetic parameters as dependent variables, with the abundance of YOY brown trout and mussel population size, and the abundance of 1 + brown trout and mussel population size, respectively, as independent variables. Mean abundance of YOY brown trout and 1 + brown trout living sympatrically with mussel populations were included in these regressions. Mussel populations with a former distribution of anadromous sea-trout and Atlantic salmon populations, but where barriers had cut-off their migration route between the Baltic Sea and the mussel populations, were not included in these analyses. Atlantic salmon does not exist in these areas today, while brown trout abundances are dramatically reduced when only tributary resident brown trout populations can spawn upstream of the migration obstacles. Thus, if the genetic composition of mussels is influenced by their host fish abundances as we hypothesized, the present brown trout abundances are not reflecting their historical abundances, when they were potentially influencing the genetic diversity of these long-lived mussels, given that the majority of mussels that we find today were recruited before the migration obstacles were constructed.

Mussel population sizes were estimated based on the Swedish standard survey protocol for the freshwater pearl mussel. In addition to the stepwise regressions, mussel population size from all rivers were also related to observed (*H*_O_) and expected (*H*_E_) heterozygosity, and inbreeding coefficient (*F*_IS_) using linear regression. Lastly, mussel population sizes were also related to the genetic variables within each drainage, using linear regressions.

## Results

### Genetic diversity

The number of alleles (*N*_A_) were between 13 (Mie and Dal) and 42 (Not). Nine out of 17 populations did not exhibit any private alleles (*N*_PA_), while the remaining ranged from one to four except in the population Not which had 11 private alleles. A large number of sampling localities showed significant deviations from Hardy–Weinberg expectation, manifested as deficit of heterozygotes. Inbreeding coefficient values (*F*_IS_) were positive for all populations, ranging from 0.02 (Bra) in the southernmost populations to 0.62 (Alg) in the northernmost populations. All inbreeding coefficient values were statistically significant (p < 0.001). The expected heterozygosity (*H*_E_) ranged between 0.18 and 0.64, while the observed heterozygosity (*H*_O_) ranged between 0.08 and 0.56. The expected and observed heterozygosity’s both had their highest values in the southernmost drainages (Table 1).

### Genetic differentiation

**Figure 2:**
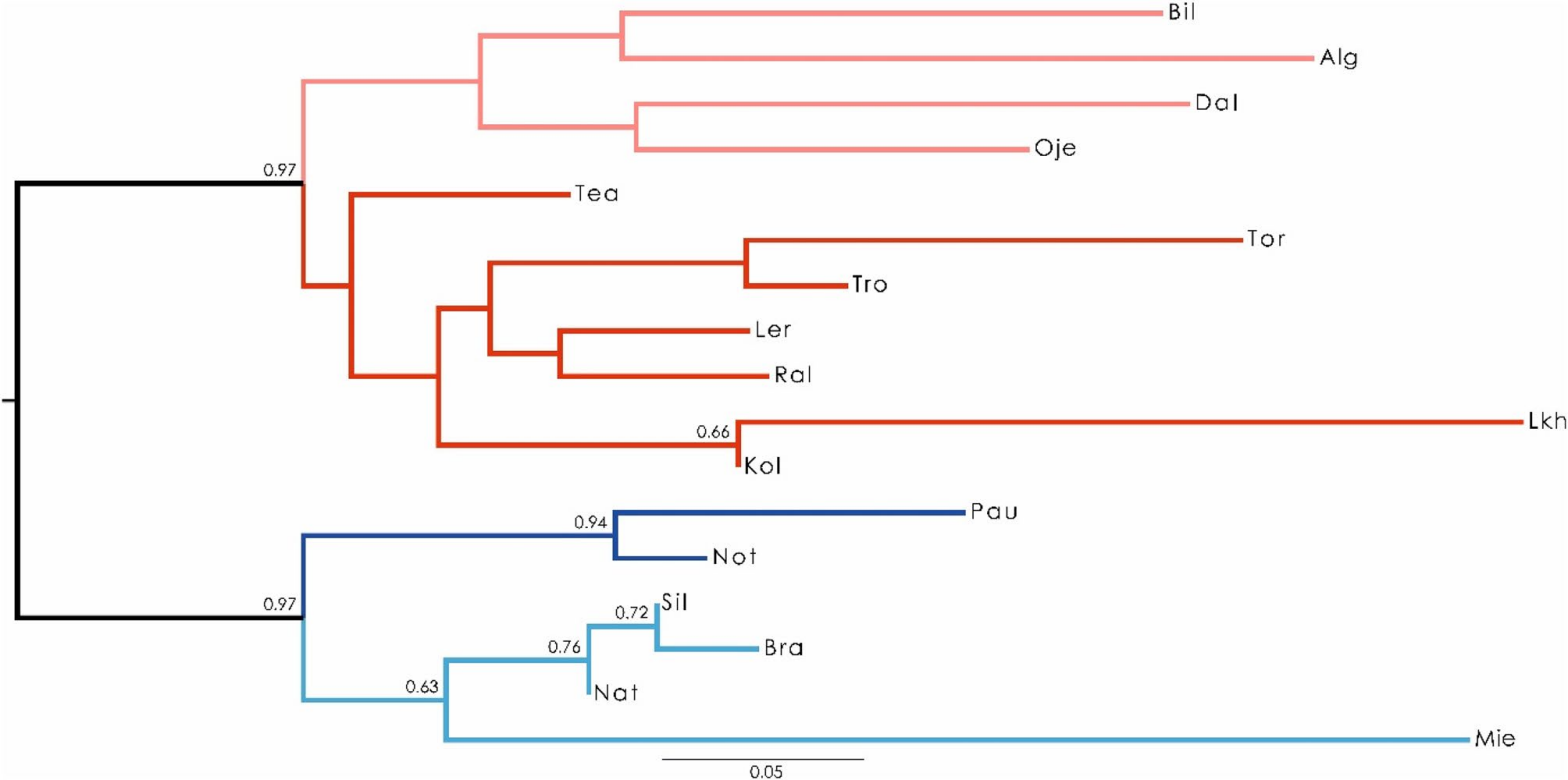
*F*_ST_-based Neighbor-Joining tree representing the freshwater pearl mussel populations analysed (bootstrap values above the branches).

The (*F*_ST_ based) Neighbour-Joining tree (NJ-*F*_ST_) (Fig. 2) reveals a first split into two well supported clades, one including the populations within the northern Göta älv and Norrström drainages and the other restricted to the southern populations from the Emån, and the Mie, Bra, Sil and Nat drainages. The latter is further subdivided in two additional supported groups, the populations in Emån drainage in one group and the Mieån, Bräkneån, Silletorpsån and Nättraån drainages in the second group (Figs. 1 and 2).

**Figure 3:**
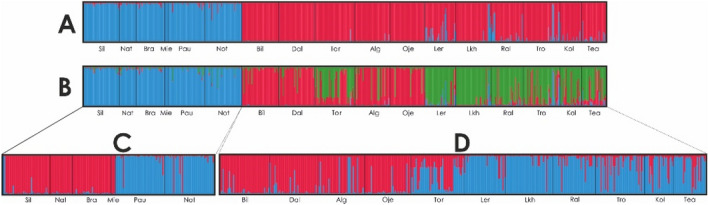
Results of the STRUCTURE Bayesian clustering analysis on freshwater pearl mussel populations: (**A**) with the most likely number of clusters (K = 2); (**B**) with the second most likely number of clusters (K = 3); (**C**,**D**) additional clustering analyses within each of the major clades identified in (**A**), both with the most likely number of clusters (K = 2). Each individual is represented by a vertical bar in K colored segments with the length of each bar being proportional to the estimated membership coefficient. Black lines separate individuals from different populations.

Plots of ΔK^35^ based on the first STRUCTURE analysis (K = 1–20) indicated that two is the most likely number of clusters present in the full dataset (Sup. Fig. 1). The two clusters correspond to the major clades obtained on the NJ- *F*_ST_ tree (Figs. 2 and 3A). All populations presented a very high individual assignment to both clusters (> 85%). The second best K value was obtained for K = 3 that further divides cluster 2 in two additional groups (Fig. 3B; Sup. Fig. 1).

**Figure 4:**
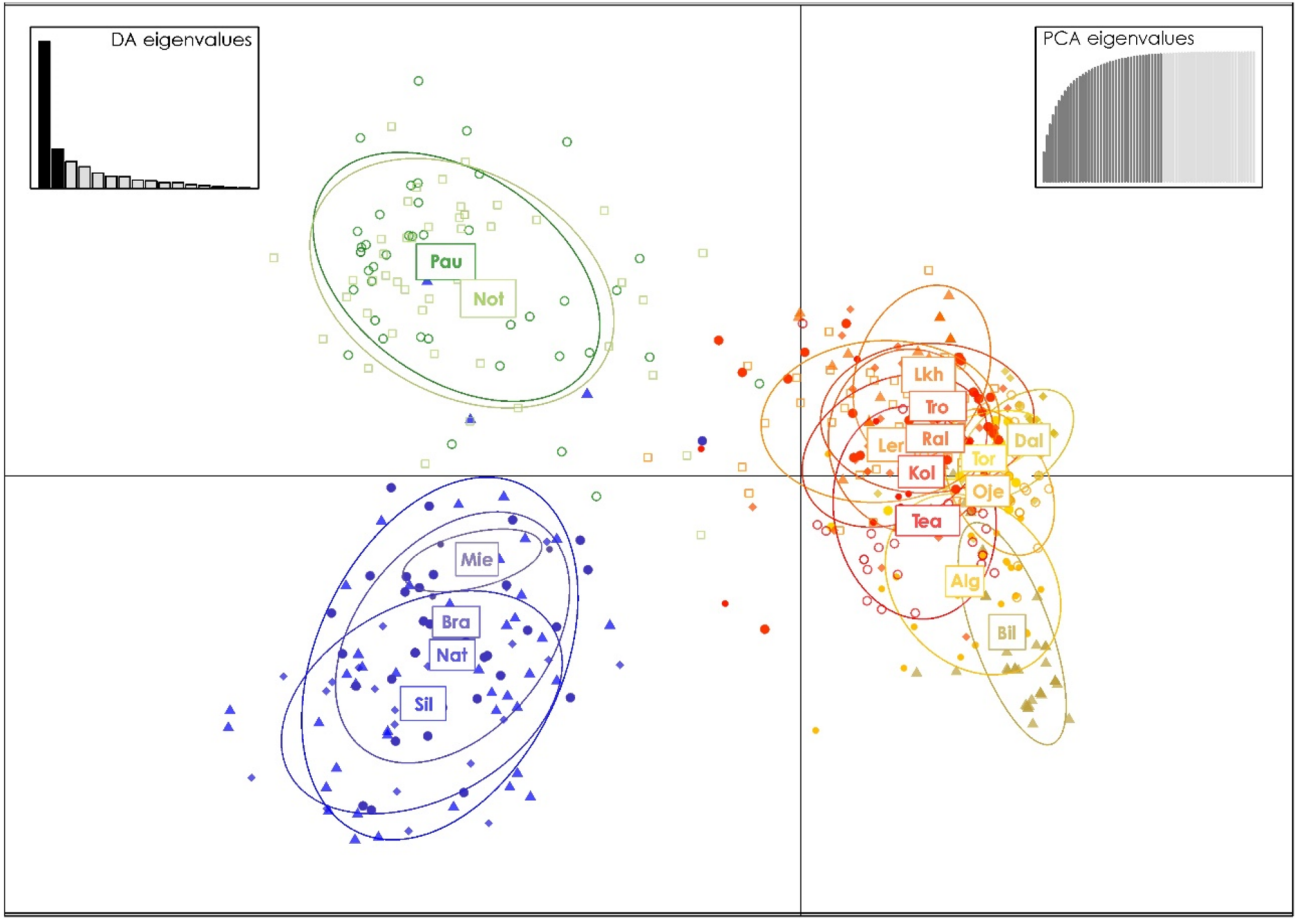
Discriminant Analysis of Principal Components (DAPC) using microsatellite loci of the freshwater pearl mussel and three genetic clusters. The DAPC graph represents the individuals as dots and the clusters as inertia ellipses.

The results of the additional STRUCTURE analyses within the clusters in the first run (K = 2) identified two additional groups, i.e. one corresponding to Sil, Nat, Bra and Mie populations and the other to Pau and Not populations (Fig. 3C; Sup. Fig. 2). Two groups were also found for the second major cluster of the first STRUCTURE analysis (Fig. 3D; Sup. Fig. 3). The DAPC analysis structured the dataset in three major clusters: one with Pau and Not, a second with Sil, Nat, Bra and Mie, and a third with all remaining populations (Fig. 4). All the population structures tested by AMOVA resulted in similar results with a higher percentage of the variation explained among groups. The remaining variation was mainly explained within populations. The structure tested for the two obtained clusters for all populations (Figs. 2 and 3A) produced the higher among group variation (Table 2).

**Table 2:** Analyses of molecular variance (AMOVA) measured among studied populations of the freshwater pearl mussel between and within clusters identified in STRUCTURE, NJ and DAPC.

Structure tested	Source of variation	% variation	Fixation indices
K = 2 STRUCTURE/NJ	Among groups	67.4	0.674
	Among populations within groups	2.8	0.086
	Within populations	29.8	0.702
K = 3 STRUCTURE	Among groups	57.4	0.574
	Among populations within groups	3.4	0.081
	Within populations	39.1	0.609
K = 3 DAPC	Among groups	65.5	0.655
	Among populations within groups	2.67	0.077
	Within populations	31.8	0.682
4 groups STRUCTURE	Among groups	56.3	0.563
2 within Cluster 1 +	Among populations within groups	3.1	0.071
2 within Cluster 2	Within populations	40.6	0.594

All results were statistically significant (p < 0.001).

Pairwise comparisons of genetic differentiation (Pairwise *F*_ST_) evidenced a well-structured population pattern, revealing a lower differentiation within geographically close southern mussel populations sustained by Atlantic salmon and Sea-migrating brown trout. The exception was the Mie population, which showed a high differentiation from the geographically close drainages Sil, Nat and Bra, and the highest differentiation to all other mussel populations. The genetic differentiation was generally high to extremely high within and among the main drainages where the mussel populations were sustained by tributary resident brown trout populations (Table 3).

**Table 3:** Pairwise estimates of *F*_ST_ (below diagonal) between all populations sampled for the freshwater pearl mussel.

	Sil	Nat	Bra	Mie	Pau	Not	Bil	Dal	Tor	Alg	Oje	Ler	Lkh	Ral	Tro	Kol
Nat	0.0032															
Bra	**0.0152**	**0.0275**														
Mie	**0.2634**	0.2191	0.2549													
Pau	**0.2368**	**0.2236**	**0.2474**	**0.3807**												
Not	**0.1984**	**0.1869**	**0.1992**	**0.3563**	**0.108**											
Bil	**0.4184**	**0.4237**	**0.4715**	**0.6176**	**0.5333**	**0.472**										
Dal	**0.4642**	**0.4799**	**0.5187**	**0.6571**	**0.5201**	**0.4497**	**0.3792**									
Tor	**0.4894**	**0.5308**	**0.5460**	**0.7765**	**0.559**	**0.4617**	**0.4339**	**0.4404**								
Alg	**0.4258**	**0.4350**	**0.4710**	**0.6120**	**0.5285**	**0.4757**	**0.3024**	**0.3164**	**0.5412**							
Oje	**0.4196**	**0.4439**	**0.4793**	**0.6473**	**0.5307**	**0.4471**	**0.3456**	**0.2275**	**0.3471**	**0.3152**						
Ler	**0.2466**	**0.253**	**0.2834**	0.4134	**0.3896**	**0.2989**	**0.2994**	**0.3558**	**0.3022**	**0.3622**	**0.2673**					
Lkh	**0.4665**	**0.5011**	**0.5243**	**0.7599**	**0.5435**	**0.4554**	**0.5608**	**0.5353**	**0.4883**	**0.6041**	**0.5039**	**0.3434**				
Ral	**0.3168**	**0.3312**	**0.3708**	0.5065	**0.4453**	**0.3443**	**0.3165**	**0.2834**	**0.2419**	**0.3778**	**0.2237**	**0.0951**	**0.3685**			
Tro	**0.3632**	**0.3792**	**0.4084**	0.6049	**0.4474**	**0.3597**	**0.3769**	**0.3531**	**0.1466**	**0.4055**	**0.2698**	**0.1400**	**0.3266**	**0.1554**		
Kol	0.3282	0.3274	**0.3818**	0.5677	**0.4149**	**0.3293**	**0.3611**	**0.2821**	**0.2565**	0.4113	**0.3013**	0.2023	0.2082	0.1392	0.1485	
Tea	**0.2925**	**0.2699**	**0.3328**	0.4744	**0.3765**	**0.3127**	**0.2246**	**0.3225**	**0.3100**	**0.2675**	**0.3083**	**0.1645**	**0.3337**	**0.1964**	**0.1860**	0.1362

In bold significant values after sequential Bonferroni correction (p < 0.004).

No migration was detected between each of the major cluster using BAYESASS. However, within cluster one, high migration rates were detected from Bra to Sil, Mie to Sil, and Not to Nat populations. Within cluster two, three migration events were also detected, i.e. from Tro to Tor, Kol to Tro, and from Oje to Dal (Table 4). The analysis of recent genetic bottlenecks only revealed a significant statistic value for the Nat population (Suppl. Table 1).

**Table 4:** Means of the posterior distributions of migration rates derived from BAYESASS for the freshwater pearl mussel between and within clusters identified in STRUCTURE analyses; m[*i*][*j*] = fraction of individuals in population *i* that are migrants derived from population *j* (see Table 1 for population codes).

Between clusters STRUCTURE: K = 2	Population *j*										
	Cluster 1	Cluster 2									
**Population i**											
Cluster 1	*0.997*	0.003									
Cluster 2	0.001	*0.999*									

Within Cluster 1 All populations	Sil	Nat	Bra	Mie	Pau	Not					
**Population i**											
Sil	*0.940*	0.014	0.014	0.012	0.012	0.008					
Nat	0.016	*0.898*	0.045	0.013	0.015	0.013					
Bra	**0.250**	0.007	*0.715*	0.012	0.007	0.008					
Mie	**0.166**	0.027	0.032	*0.721*	0.027	0.027					
Pau	0.007	0.015	0.025	0.026	*0.914*	0.012					
Not	0.009	**0.239**	0.027	0.009	0.036	*0.678*					

Within Cluster 2 All populations	Bil	Dal	Tor	Alg	Oje	Ler	Lkh	Ral	Tro	Kol	Tea
Bil	*0.885*	0.006	0.008	0.007	0.014	0.006	0.006	0.006	0.006	0.049	0.007
Dal	0.006	*0.717*	0.007	0.006	**0.185**	0.006	0.006	0.006	0.011	0.045	0.006
Tor	0.007	0.007	*0.882*	0.006	0.011	0.005	0.006	0.006	0.043	0.017	0.011
Alg	0.014	0.007	0.024	*0.853*	0.013	0.006	0.006	0.008	0.038	0.019	0.011
Oje	0.007	0.010	0.011	0.013	*0.895*	0.006	0.007	0.006	0.029	0.010	0.007
Ler	0.008	0.008	0.026	0.008	0.008	*0.822*	0.012	0.008	0.037	0.052	0.011
Lkh	0.006	0.008	0.007	0.006	0.006	0.006	*0.873*	0.008	0.060	0.006	0.012
Ral	0.007	0.008	0.015	0.007	0.009	0.008	0.008	*0.840*	0.043	0.024	0.030
Tro	0.006	0.006	**0.170**	0.011	0.007	0.012	0.008	0.006	*0.735*	0.017	0.021
Kol	0.009	0.009	0.019	0.009	0.009	0.009	0.012	0.009	**0.105**	*0.781*	0.029
Tea	0.013	0.009	0.039	0.010	0.013	0.008	0.009	0.009	0.062	0.009	*0.817*

*m* rates within samples are on the diagonal (italicized values). Higher migration rates are shown in bold.

### Genetic diversity and the relation to fish and mussel characteristics

The results of the sea-migratory vs. tributary resident life history of the host fish on genetic diversity and inbreeding coefficient between mussel populations showed that the observed (*H*_O_) and expected (*H*_E_) heterozygosity were both higher in mussel populations with sea-migrating brown trout / Atlantic salmon as host fish (*H*_O_: 0.48 ± 0.086; *H*_E_: 0.54 ± 0.12) than in mussel populations with tributary resident brown trout population as host fish (*H*_O_: 0.23 ± 0.13; *H*_E_: 0.36 ± 0.12) (Mann–Whitney U-tests; *H*_O_: p = 0.003; *H*_E_: p = 0.02). The inbreeding coefficient (*F*_IS_) were significantly lower in mussel populations with sea-migrating brown trout / Atlantic salmon as host fish (*F*_IS_: 0.13 ± 0.063) than in mussel populations with tributary resident brown trout population as host fish (*F*_IS_: 0.43 ± 0.16) (Mann–Whitney U-test, p = 0.003).

**Figure 5:**
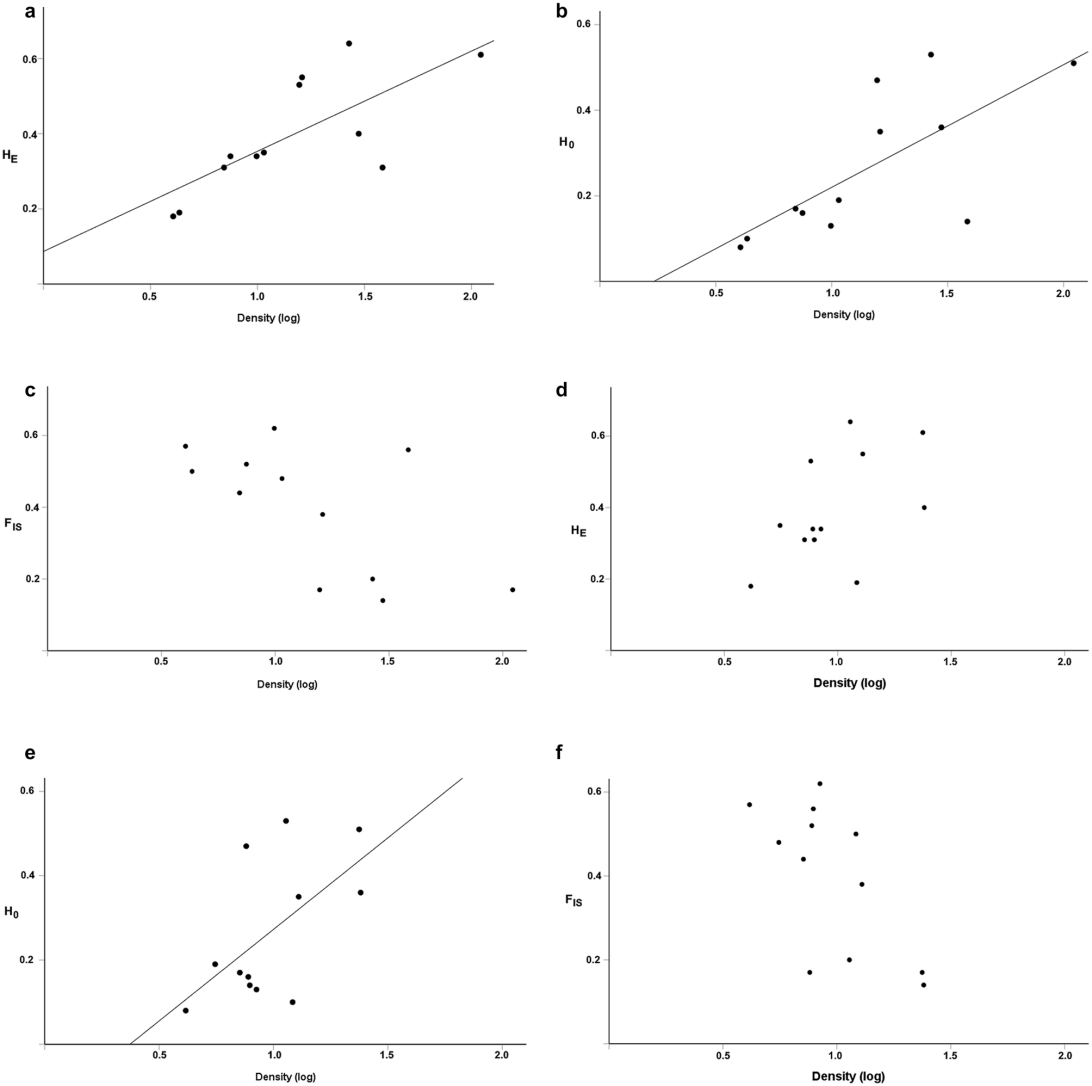
Mean expected heterozygosity (*H*_E_), mean observed heterozygosity (*H*_O_) and mean inbreeding coefficient (*F*_IS_) as a function of abundance of young-of-the-year fish (YOY) (**a**–**c**) and of fish older than young-of-the-year (OLD) (**d**–**f**) (log individuals 100 m^−2^), respectively.

To test if brown trout abundance and the size of mussel populations were influencing the observed (*H*_O_) and expected (*H*_E_) heterozygosity, and inbreeding coefficient (*F*_IS_), the abundance of YOY brown trout and mussel population size, and the abundance of brown trout older than one year and mussel population size, respectively, were tested in stepwise regressions. Mussel population size did not have any significant effect on the observed (*H*_O_) and expected (*H*_E_) heterozygosity, or the inbreeding coefficient (*F*_IS_), and was excluded in all multiple regressions, both when regressed with the abundance YOY brown trout and the abundance of brown trout older than one year (p > 0.05). There was however a significant positive relationships between the abundance of YOY brown trout and observed heterozygosity (*H*_O_) (Stepwise regression, y = 1.34x + 0.81, r^2^ = 0.37, n = 12, p = 0.021), and between the abundance of YOY brown trout and expected heterozygosity (*H*_E_) (Stepwise regressions, y = 1.38 x + 0.80, r^2^ = 0.37, n = 12, p = 0.022), but no relationship between the abundance of YOY brown trout and inbreeding coefficient (*F*_IS_) (p = 0.059). For older brown trout, there were no significant relationships between the abundance of brown trout older than one year or mussel population size and observed heterozygosity (*H*_O_) (Stepwise regression, p = 0.14), between the abundance of brown trout older than one year and expected heterozygosity (*H*_E_) (Stepwise regression, p = 0.13), or between the abundance of brown trout older than one year and inbreeding coefficient (*F*_IS_) (Stepwise regression, p = 0.18) (Fig. 5a–f).

The mussel population sizes from all rivers were also related to the genetic variables, but there were no significant relationships between mussel population size and the expected heterozygosity (*H*_E_) or observed heterozygosity (*H*_O_) or the mean inbreeding coefficient (*F*_IS_) (p > 0.05). Analyses were also performed for populations within each drainage. There was a significant relationship between population size and the expected heterozygosity (*H*_E_), for the Göta Älv drainage (Regression, y = 0.09 x − 0.017, r^2^ = 0.54, n = 8, p < 0.024), but not between any other genetic variables for any drainage.

## Discussion

The present study provides new information on genetic structure among and within drainages, and in relation to variation in life history among host fish populations in southern Scandinavia, with importance for the conservation of unionoid mussels. The generally drainage-independent genetic differentiation was genetically structured into two main clusters, one including the Northern drainages and the other with the most Southern ones (Fig. 1), identified by both the initial STRUCTURE (best K = 2; Fig. 3A) and *F*_ST_ based Neighbour-Joining tree (Fig. 2) analyses. A third cluster was detected within the Northern group drainages (second best K = 3; Fig. 3B. Finally, a forth cluster was detected on the Southern group drainages, by the DAPC analyses (Fig. 4) and on the additional STRUCTURE analysis performed (best K = 2; Fig. 3C). The population-group structure tests revealed by AMOVA indicated a higher percentage of the variation explained among the tested groups. Although the higher percentage of the variation among groups was for the first division into two population-groups, the other two substructures found revealed a similar level of variation among population-groups. Thus supporting the division of the entire dataset into four genetically differentiated population-groups, here recognized as four main conservation units^43^, which may be the result of isolation, vicariance and postglacial colonization^20,44^. The genetic patterns were also related to the migratory/resident life history of the host fish, where differentiation was high where mussels are sustained by tributary resident host fish, but lower among mussel populations sustained by sea-migrating host fish. Additionally, the genetic diversity was high in the southernmost mussel populations sustained by sea-migrating host fish, and lower in mussel populations sustained by tributary resident host fish. In particular, the present study showed that not only Atlantic salmon, but also sea-migrating brown trout, seem to provide a base for high genetic diversity for their sympatric mussel populations, and even so in rivers with low mussel population size. In contrast, mussel population size in rivers sustained by tributary resident brown trout appeared to have a positive effect on genetic diversity. We also showed that brown trout abundance might positively affect genetic diversity of mussel populations. The results of our study thus suggest a combined influence of mussels and host fish on genetic structure on unionoid mussel populations, which highlights the complex interactions that affect these host dependent and threatened mussels.

### Genetic differentiation

The large-scale genetic structure showed a partly geographic drainage-independent genetic structure. The four geographically close southernmost main drainages Silletorpsån, Nättrabyån, Bräkneån and Mieån, which were considered as one conservation unit, indicated a drainage-independent genetic structure, since these populations generally had low genetic differentiation. The populations in the Emån main drainage, on the other hand, indicated a drainage-dependent genetic structure, often considered as a result of isolation and vicariance^43^. The genetic structure of the mussel populations in the Göta älv and Norrström main drainages were mainly drainage-independent, since four populations in the Göta älv drainage were clustered together with the three populations in the Norrström drainage. The four remaining populations in the Göta älv drainage, which were clustered in a separate clade, are however all situated in the same sub-drainage. Genetic drift caused by founder effects or bottleneck processes in this sub-drainage may explain this pattern. The remaining population in this sub-drainage, the Tor population, was grouped in the other clade, mostly due to the presence of hybrid individuals between the predominant lineages from the Norrström and Göta älv drainages. The genetic structure in the Göta älv and Norrström main drainages, as for the populations in the other main drainages, could be related to a combination of vicariance and isolation^43^. It might also be related to postglacial colonization events^45^, both by mussels and host fish of different linages. Two main lineages of Atlantic salmon seem to inhabit the rivers in Sweden, while colonization by brown trout seems more diverse, based on the mixed distribution of several Atlantic lineages in glaciated areas of Scandinavia^46^, with potential effects on the genetic composition of the freshwater pearl mussel. In contrast, the mussel is considered to belong to one genetic population over the large geographic distribution area in North America, potentially because of fewer colonization events compared to northern Europe, and in combination with a slow rate of molecular evolution^44^.

Additionally, the patterns of the genetic differentiation may also be explained by the movement and migration patterns of the host fish. In fact, the genetic differentiation between mussel populations can be low between populations sustained by Atlantic salmon, also over large geographic areas^10^. Our results also added the fact that this pattern may not be species specific, since mussels using sea-migrating brown trout were also associated with low genetic differentiation among their populations. In contrast, the differentiation was high among and within drainages where tributary resident brown trout are the host. The genetic differentiation may thus not only be structured among drainages or host fish species, but also depending on the migratory/residency behavior of the host fish. There are in fact reasons to believe that the migratory behavior of host fish influence the genetic structure of mussel populations. This is because the passive movement of unionoid mussels are highly dependent on the mobility of their host fish, why the patterns of their genetic differentiation may also be related to the genetic differentiation of their host fish. The results by^11^ strengthen this theory, since they showed that tributary resident brown trout are highly genetically differentiated, while sea-migrating brown trout show lower genetic differentiation among their populations.

The freshwater pearl mussel is a long-lived species with a long generation time, and thus has a slow rate of molecular evolution, which decreases the potential for large differentiation among their populations^44^. The large genetic differentiation can, however, be higher than that of their host fish^11^, but can also be related to the movement patterns of the host fish^47–49^. One explanation may be that establishment of some mussel populations go through a founder effect, driven by their host-dependent movement. When mussels colonize new locations, this is probably done through the passive movement of few mussel larvae by few host fish individuals. Few mussels with only a small proportion of the genetic pool from the source population may thus be introduced into new locations. When a new mussel population is established and increase in size, the population could then be genetically differentiated compared to the source population. For mussel populations sustained by tributary resident brown trout, normally having low rates of movement in inland areas with low connectivity^11^, the genetic migration rates are probably low as found in our investigation. In contrast, the results of our study revealed that sea-migrating brown trout has the potential to connect mussel populations among drainages. However, there was one exception to this pattern, the Mie mussel population, which had a highest genetic differentiation compared to the geographically close mussel populations sustained by sea-migrating brown trout. Genetic drift in this extremely small mussel population probably caused this pattern.

### Genetic diversity

The observed genetic diversity was generally low compared to those from mid-Sweden^45^, especially in populations sustained by tributary-resident trout populations. Such patterns have also been observed in other regions, where the genetic diversity was high in mussel populations sustained by Atlantic salmon, but not in most mussel populations sustained by resident brown trout^10^. This may be a result of the differentiation of the inland mussel populations with low connectivity for their brown trout host fish^11^. Interestingly, sea-migrating brown trout was also associated with high genetic diversity for their sympatric mussel populations in our investigation. Thus, high genetic diversity of mussel populations may not only depend on specific species^24^, but their sea-migratory life history, i.e. Atlantic salmon and sea-migrating brown trout, where high connectivity seem to provide a base for genetic diversity of mussel populations.

The lowest genetic diversity and highest inbreeding coefficients were observed in one sub-drainage to Göta älv. This pattern may be a consequence of founder and bottleneck effects caused by low connectivity of inland mussel populations through their resident brown trout hosts. Furthermore, the genetic diversity in the Göta älv drainage was positively related to mussel population size. This relationship should be interpreted with caution, given that we found this relationship in only one drainage, even if there is evidence of a positive relationship between genetic diversity and population size for many species^50–51^. We did not find this relationship when mussel populations sustained by sea-migrating brown trout and Atlantic salmon was included in the analyses. In fact, high genetic diversity and low inbreeding was found in mussel populations with low population sizes when sustained by sea-migrating brown trout. High gene flow may be facilitated by the high connectivity within and between drainages and counteract genetic drift, sustained by the large migratory distances and straying behavior of the sea-migrating brown trout and salmon connecting the mussel populations. This argumentation is also strengthened by the fact that sea-migrating brown trout often have high genetic diversity, in contrast to the low genetic diversity for resident brown trout^11^.

There was one exception of a mussel population (Mie) which had lower genetic diversity and higher differentiation compared to the three geographically close mussel populations sustained by sea-migrating brown trout. Genetic drift may explain some of these differences in this extremely small populations, but also the fact that the population may partly by sustained by lake-migrating brown trout. Also, even if these four mussel populations had high genetic diversity, the mussel population in Nat sustained by sea-migrating brown trout seemed to have gone through a bottleneck. This may possibly be a result of the low population size, which shows that also these populations are sensitive and need prioritization in management.

Interestingly, brown trout abundance, including tributary-resident and sea-migrating populations, appears to positively affect the genetic diversity of mussel populations. Many brown trout individuals over a large distribution may in fact increase the potential for a high number of spawning mussel individuals to infect their host fish. This should result in high infection rates of the parasitic mussel larvae with a diverse genetic composition, sustaining the genetic diversity of the benthic mussel population. Host fish abundance can thus be one key to understand population genetics also of other unionoid mussel species. Such relations between host fish abundance and genetic diversity may add to factors making the unionoid mussels highly threatened^52^. These results also highlight the complex interactions and factors structuring the genetic composition of threatened mussels with a parasitic life stage.

### Conservation implications

The present study shows that population genetic studies can be of high importance when protecting and managing threatened unionoid mussels. We suggest that the freshwater pearl mussel populations in the study area should be divided into four conservation units^43^. This represents two conservation units mixed over the Göta älv and Norrström main drainages, one conservation unit in the Emån main drainage, and one conservation unit in the four geographically close southernmost drainages sustained by sea-migrating brown trout. We also suggest considering resident/migratory life history of the host when designing management strategies and conservation programs.

The results of our study also call for attention on conservation of population sizes and abundances of the mussels^21^ and their host fish. This implies that there seem to be a special need to protect mussel populations sustained by tributary-resident brown trout populations from further decline^10^. In such areas, the high frequency of fragmentation by migration obstacles (i.e. dams and hydropower plants) reduce the connectivity for the host fish^10^, and is a probably a large threat to gene flow between mussel populations^21^. In contrast, even small mussel populations can be genetically diverse when sustained by sea-migrating brown trout, even if their density is also important for genetic. In addition, host fish abundance appear to positively influence genetic diversity, why managers should make sure that fish abundance stays intact over time.

Given the decreasing population sizes and extinctions of the freshwater pearl mussel, translocation and propagation are often considered^53^. According to the results from our study and others^54^, we suggest that population genetic structure assessments are vital before such activities are performed. Information about conservation units is a first step to evaluate which mussel strains to use in translocation and propagation programs. Careful investigations of the genetic structure of sub-populations should however be prioritized, given the high genetic differentiation among mussels from different tributaries, especially for mussels sustained by tributary-resident brown trout^21^. In addition, co-evolutionary considerations should also be considered, since disruption of locally adapted mussels and host fish strains living in sympatry can result in changes in recruitment success^55–57^. Investigations of the genetic structure should thus also be applied to geographically close mussel populations sustained by sea-migrating brown trout and Atlantic salmon, even with their generally low genetic differentiation and high genetic diversity. In summary, our results highlight the complex management for threatened species with life cycles including a parasitic stage on a host.

## Supplementary information

Supplementary Figures.

Supplementary Table.

## Data Availability

The datasets generated during and/or analyzed during the current study are available from the corresponding author on reasonable request.
