# Supplementary material for: The genetic diversity and differentiation of mussels with complex life cycles and relations to host fish migratory traits and densities
Source: Sci Rep. 2020 Oct 15;10:17435. doi: 10.1038/s41598-020-74261-z (PMC7567107; doi:10.1038/s41598-020-74261-z)
Supplement: Supplementary file 1 — Supplementary Figures. [file 41598_2020_74261_MOESM1_ESM.docx]

**Supplementary Figures**

**The genetic diversity and differentiation of mussels with complex life cycles and relations to host fish migratory traits and densities**

Martin Österling^a^, Manuel Lopes-Lima^b^, Elsa Froufe^b^, Amra H Hadzihalilovic^a^, Björn Arvidsson^a^.

^a^Department of Environmental and Life Sciences – Biology, Karlstad University, Universitetsgatan 2, 651 88 Karlstad, Sweden

b CIIMAR/CIMAR - Interdisciplinary Centre of Marine and Environmental Research, University of Porto, Terminal de Cruzeiros do Porto de Leixões, Av. General Norton de Matos s/n, 4450-208 Matosinhos, Portugal

Corresponding author: E-mail address: [martin.osterling@kau.se](mailto:martin.osterling@kau.se) ORCID: 0000-0001-6758-5857; Tel: +46 54 7001802.

**Supplementary Figure 1.** Plots generated in STRUCTURE Harvester for all populations showing: TOP-LEFT - the Evanno's delta K statistic; TOP-RIGHT - the mean rate of change of the likely distribution L’(K); BOTTOM-LEFT – the mean absolute value of the 2^nd^ order rate of change of the likelihood distribution |L’’(K)|; and BOTTOM-RIGHT - the mean log likelihood of the data [L(K)].

S**upplementary Figure 2.** Plots generated in STRUCTURE Harvester for the Southern Populations showing: TOP-LEFT - the Evanno's delta K statistic; TOP-RIGHT - the mean rate of change of the likely distribution L’(K); BOTTOM-LEFT – the mean absolute value of the 2^nd^ order rate of change of the likelihood distribution |L’’(K)|; and BOTTOM-RIGHT - the mean log likelihood of the data [L(K)].

**Supplementary Figure 3.** Plots generated in STRUCTURE Harvester for the Northern Populations showing: TOP-LEFT - the Evanno's delta K statistic; TOP-RIGHT - the mean rate of change of the likely distribution L’(K); BOTTOM-LEFT – the mean absolute value of the 2^nd^ order rate of change of the likelihood distribution |L’’(K)|; and BOTTOM-RIGHT - the mean log likelihood of the data [L(K)].

**
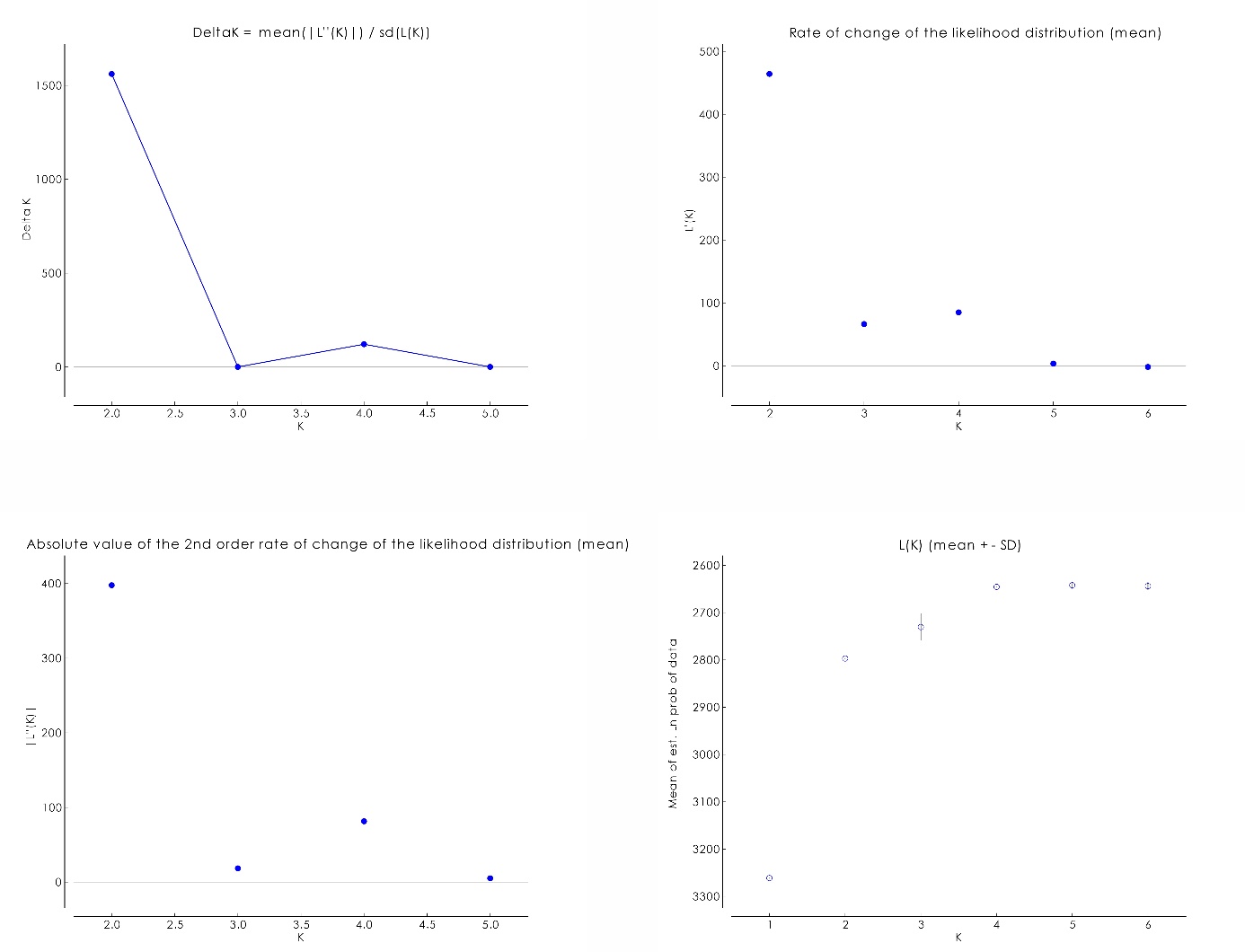
**

**Supplementary Figure 1.**

**
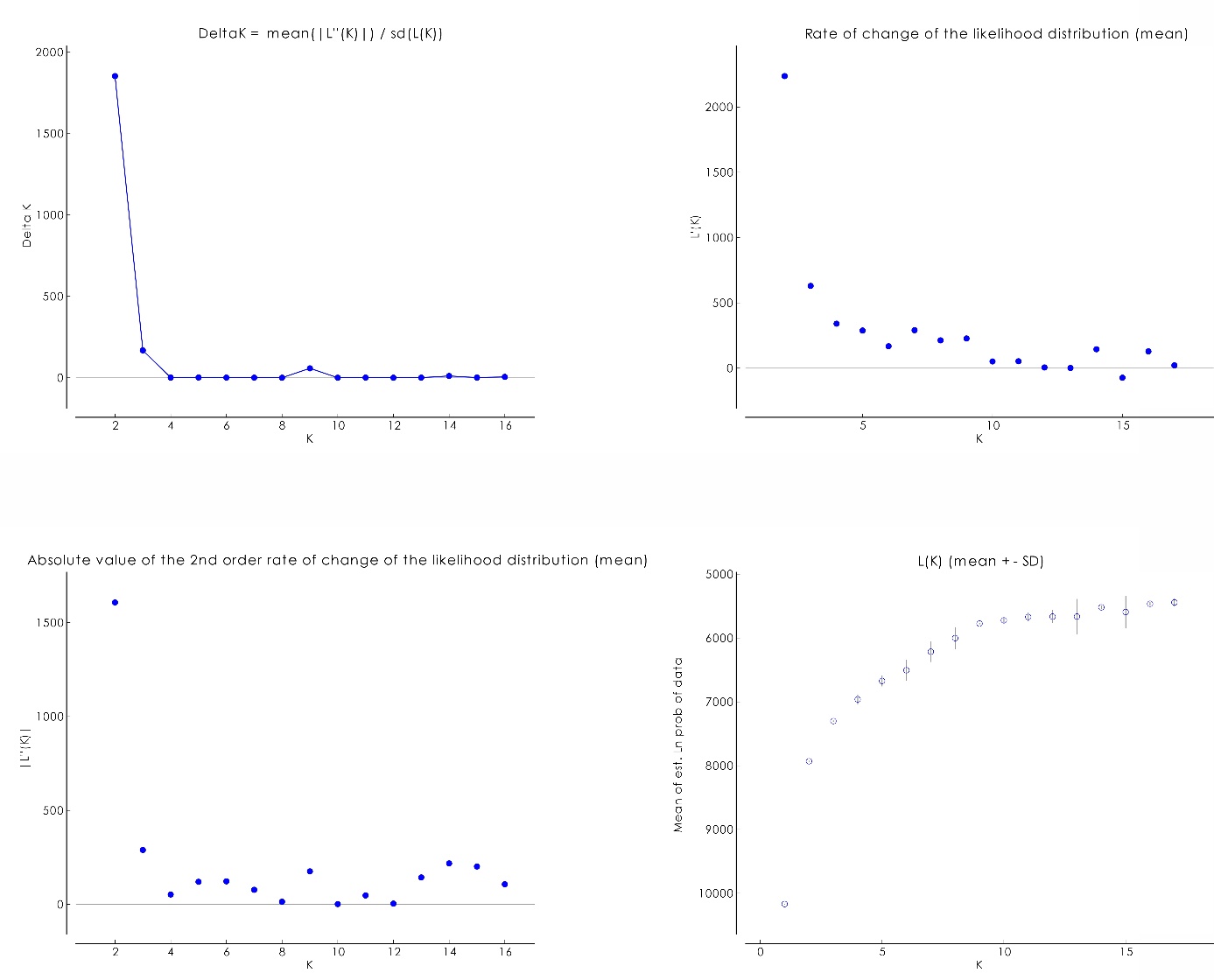
**

**Supplementary Figure 2.**

**
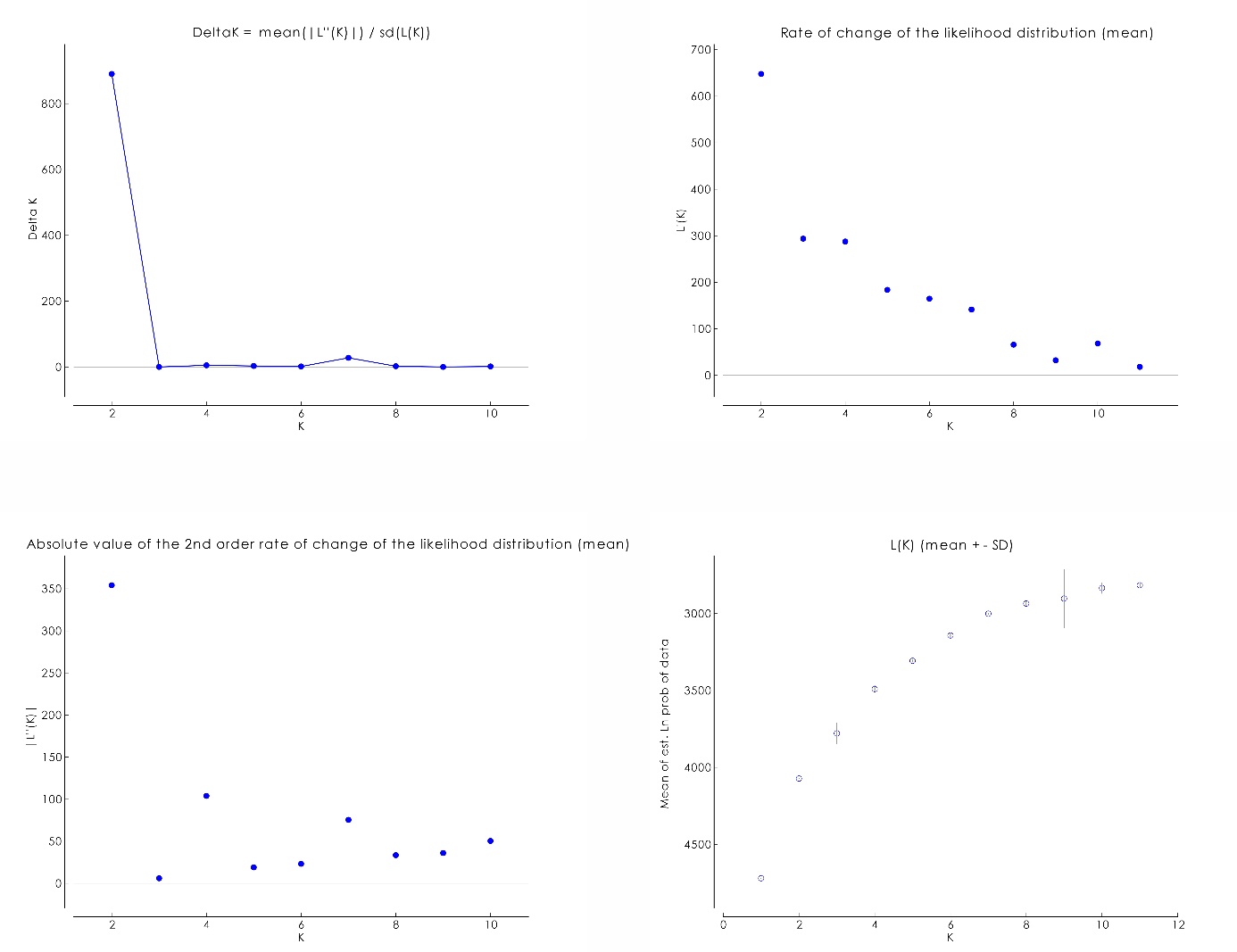
**

**Supplementary Figure 3.**
