# Supplementary material for: The genetic diversity and differentiation of mussels with complex life cycles and relations to host fish migratory traits and densities
Source: Sci Rep. 2020 Oct 15;10:17435. doi: 10.1038/s41598-020-74261-z (PMC7567107; doi:10.1038/s41598-020-74261-z)
Supplement: Supplementary file 2 — Supplementary Table. [file 41598_2020_74261_MOESM2_ESM.docx]

**Supplementary Table**

**Supplementary Table 1 .** Analysis of recent genetic bottlenecks in *Margaritifera margaritifera* estimated for 6 microsatellite loci in 17 populations using the sign-rank Wilcoxon test of the mutation–drift equilibrium under a two-phased model of mutation (TPM) (H_0_: H_e_=H_eq_ - population at mutation-drift equilibrium; H_1_: H_e_ > H_eq_ - excess gene diversity indicating recent bottleneck). Significance supporting a recent population bottleneck is shown in bold. (see Table 1 for population codes).

|  | Populations: Wilcoxon test (He > Heq) | | | | | | | | | | | | | | | |  |
| --- | --- | --- | --- | --- | --- | --- | --- | --- | --- | --- | --- | --- | --- | --- | --- | --- | --- |
|  | **Sil** | **Nat** | **Bra** | **Mie** | **Pau** | **Not** | **Bil** | **Dal** | **Tor** | **Alg** | **Oje** | **Ler** | **Lkh** | **Ral** | **Tro** | **Kol** | **Tea** |
| *P*-values | 0.50 | **0.008** | 0.66 | 0.95 | 0.96 | 0.72 | 0.31 | 0.06 | 0.95 | 0.31 | 0.50 | 0.71 | 1.0 | 0.42 | 0.98 | 0.95 | 0.66 |
